# Diverse Dataset for Eyeglasses Detection: Extending the Flickr-Faces-HQ (FFHQ) Dataset

**DOI:** 10.3390/s24237697

**Published:** 2024-12-01

**Authors:** Dalius Matuzevičius

**Affiliations:** Department of Electronic Systems, Vilnius Gediminas Technical University (VILNIUS TECH), 10105 Vilnius, Lithuania; dalius.matuzevicius@vilniustech.lt

**Keywords:** eyewear detection, glasses detection, eyeglasses, FFHQ dataset, object detection, public dataset

## Abstract

Facial analysis is an important area of research in computer vision and machine learning, with applications spanning security, healthcare, and user interaction systems. The data-centric AI approach emphasizes the importance of high-quality, diverse, and well-annotated datasets in driving advancements in this field. However, current facial datasets, such as Flickr-Faces-HQ (FFHQ), lack detailed annotations for detecting facial accessories, particularly eyeglasses. This work addresses this limitation by extending the FFHQ dataset with precise bounding box annotations for eyeglasses detection, enhancing its utility for data-centric AI applications. The extended dataset comprises 70,000 images, including over 16,000 images containing eyewear, and it exceeds the CelebAMask-HQ dataset in size and diversity. A semi-automated protocol was employed to efficiently generate accurate bounding box annotations, minimizing the demand for extensive manual labeling. This enriched dataset serves as a valuable resource for training and benchmarking eyewear detection models. Additionally, the baseline benchmark results for eyeglasses detection were presented using deep learning methods, including YOLOv8 and MobileNetV3. The evaluation, conducted through cross-dataset validation, demonstrated the robustness of models trained on the extended FFHQ dataset with their superior performances over existing alternative CelebAMask-HQ. The extended dataset, which has been made publicly available, is expected to support future research and development in eyewear detection, contributing to advancements in facial analysis and related fields.

## 1. Introduction

Object detection, a core component of computer vision (CV), identifies and localizes the objects within images and has broad applications in fields such as autonomous driving, surveillance, healthcare, remote sensing, and robotics [1,2,3]. Convolutional neural network (CNN)-based detectors like Faster R-CNN [4], SSD [5], and YOLO [6] have significantly enhanced accuracy and processing speed, benefiting from extensive datasets like MS COCO [7] and ImageNet [8], as well as by employing architectures that balance speed and accuracy [9,10]. More recently, transformer-based models, which excel in managing long-range dependencies and complex interactions within images, have started to outperform CNNs in object detection [11,12]. Despite these advancements, object detection in challenging conditions remains a vital area of research [13,14,15,16], with issues such as occlusions, low lighting, and transparency affecting performance [17,18,19,20]. A data-centric AI approach [21,22,23,24] emphasizes improving the quality, diversity, and representativeness of datasets rather than solely focusing on model architecture enhancements. By addressing biases, enhancing annotation accuracy, and curating data for underrepresented scenarios, this approach ensures a more robust and generalizable performance in object detection tasks. The ongoing development and refinement of specialized datasets [25,26,27], particularly for niche applications like eyewear detection where existing datasets fall short in diversity and annotation depth, are critical in advancing object detection technology and ensuring its applicability across diverse and challenging real-world situations [28].

Facial analysis has become an important area of research in CV, and it is driven by the development of large, high-quality datasets. One such dataset, the Flickr-Faces-HQ (FFHQ) dataset [29], has gained widespread use in tasks such as facial recognition, generative modeling, and facial attribute classification. FFHQ provides a diverse collection of high-resolution facial images across various ages, ethnicities, and lighting conditions, making it a valuable resource for research requiring diversity in facial appearances. However, despite its broad applicability, FFHQ lacks detailed annotations for detecting facial accessories, particularly eyewear, which presents a notable limitation for specific tasks that require the accurate identification of glasses [30,31,32].

Eyewear detection is important for a range of applications [33,34,35], including facial recognition systems [36], forensics [37] augmented reality (AR) [38], assistive technologies, and security systems [39,40]. For instance, eyewear can significantly alter facial appearance, affecting the accuracy of recognition algorithms, while AR applications benefit from a precise localization of glasses to enhance virtual try-on or personalization features [41,42,43,44,45,46]. Similarly, assistive technologies aimed at aiding visually impaired individuals in recognizing objects, including glasses, would benefit from improved detection models [47]. Security systems, especially those involving identity verification in surveillance footage, require the robust detection of eyewear to enhance the accuracy of facial recognition [48,49,50]. However, the absence of sufficient labels for glasses detection in widely used datasets such as FFHQ hinders progress in these areas.

**Figure 1:**
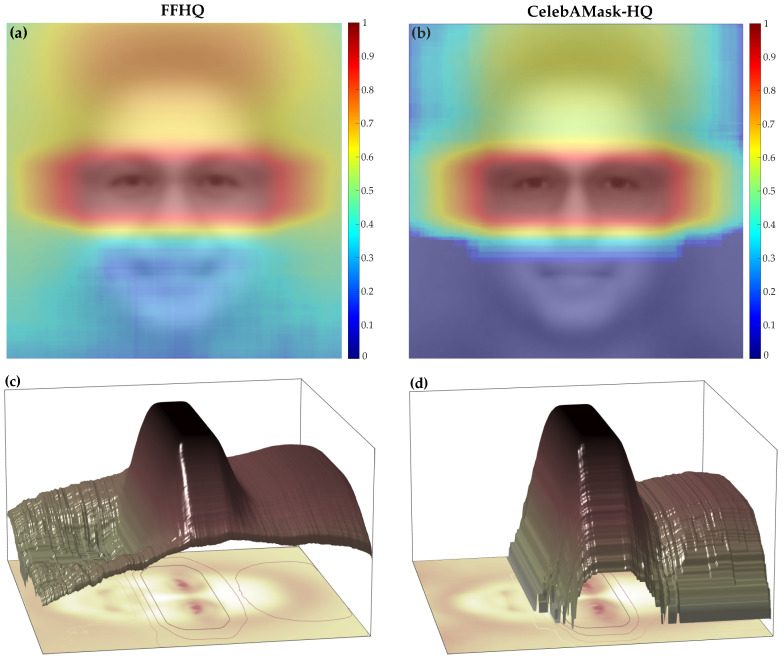
Heatmaps of the eyewear bounding box (BBox) locations in the Flickr-Faces-HQ (FFHQ) (BBox labels are the result of this research) (**a**,**c**) and CelebAMask-HQ (**b**,**d**) datasets: (**a**,**b**) 2D heatmaps; (**c**,**d**) 3D heatmaps. Both heatmaps show the bounding box locations of glasses, where the average images of individuals wearing glasses from each dataset are superimposed. The heatmaps are represented in log-scale intensity, and they show that the FFHQ dataset exhibits greater variability in the locations of glasses compared to the CelebAMask-HQ dataset. CelebAMask-HQ originally contained glasses segmentation masks that were used to generate the bounding box labels.

Currently the CelebAMask-HQ dataset [51] offers annotations for glasses in the form of pixel-wise masks (Figure 1), but it has significant limitations. Although these masks can be transformed into bounding boxes for eyewear detection, the dataset lacks sufficient diversity in terms of age, ethnic backgrounds, and general image quality compared to FFHQ [29]. CelebAMask-HQ is primarily composed of celebrity images, limiting its applicability to tasks that require a broader demographic representation. Other existing datasets similarly fall short of providing the extensive annotations required for robust eyewear detection, leaving a significant gap for training and testing ML models designed to handle variations in glasses shapes, sizes, and styles [52,53].

In addition to the limitations of existing datasets, eyewear detection presents a set of inherent challenges for CV models. Glasses come in a wide variety of shapes and styles, and their transparency introduces complexities in detection [54,55,56]. Reflections and lighting conditions may obscure glasses or cause misleading highlights, resulting in occlusions that reduce detection accuracy [57,58]. These factors create a difficult environment for traditional and deep learning models to accurately localize and identify eyewear [59], highlighting the need for a dataset that can account for these variables [60,61].

### The Novelty and Contributions of This Work

To address the limitations of the CelebAMask-HQ dataset and the challenges of eyewear detection, this work extends the FFHQ dataset by introducing new annotations for eyewear detection in the form of bounding boxes. This extension provides the first large-scale and diverse dataset specifically designed for glasses detection. The dataset significantly improves upon CelebAMask-HQ in terms of both size and diversity, offering an enriched resource for training and testing eyewear detection models. Furthermore, the use of a semi-automated labeling protocol enables efficient and accurate generation of bounding box annotations, reducing the need for extensive manual effort while maintaining annotation precision. The extended FFHQ dataset will be made publicly available, enabling further research and development in facial analysis applications.

The main objectives of this research are to (1) introduce bounding box annotations for eyewear in the FFHQ dataset, and to (2) provide baseline benchmark results for eyewear detection using the extended dataset. Detailed statistics about the dataset, including the distribution of glasses in relation to facial landmarks, object size statistics, and demographic attributes, such as age and gender, are provided. This dataset has potential applications in a wide array of fields, including facial recognition [26,62], AR [63], assistive technologies [64], fashion [65], and other systems [66,67]. By addressing the current gap in available resources for eyewear detection, this research advances the state of the art in facial analysis, particularly for applications where glasses play a critical role [68,69,70].

The following is a summary of the novel contributions and impact of this work:

- Extending the Flickr-Faces-HQ (FFHQ) dataset by providing publicly available bounding boxes of glasses (eyewear), enriching the dataset for further research in facial analysis, particularly in applications involving glasses detection.
- Introducing the first large and diverse eyeglasses detection dataset, which exceeds the only existing dataset, CelebAMask-HQ, by size and diversity, providing a richer resource for training and evaluating eyeglasses detection models.
- Proposing a protocol for the semi-automated labeling of eyewear on face images from the FFHQ dataset, significantly reducing the manual labeling effort and maximizing accuracy in bounding box annotations.
- Presenting comprehensive dataset-related statistics, including the distribution of glasses locations on face images, object (bounding box) size statistics, and distribution across various demographic attributes (e.g., age, gender, etc.).
- Performing a comparative evaluation of baseline detection algorithms for glasses detection using the extended FFHQ dataset as a benchmark to assess performance, and presenting results that establish a baseline for future research in eyeglasses detection.
- Demonstrating the effectiveness of the extended dataset by showing the improved detection results of glasses using deep learning methods on the smaller existing CelebAMask-HQ dataset.
- Publicly releasing the glasses-labeled FFHQ extension, offering a resource for the computer vision community to advance research in facial recognition, attribute classification, and other related applications where eyewear plays a role.

The structure of this paper is organized as follows: Section 2 provides an overview of the materials and methods employed in this study, detailing the FFHQ dataset extension process and annotation protocols, and it also highlights the need for improved eyewear detection in existing datasets and outlines the procedure used to extend the Flickr-Faces-HQ (FFHQ) dataset with bounding box annotations for glasses. In Section 3, the results of the study are discussed, presenting baseline detection performances using various deep learning models and comparative analysis across datasets. The effectiveness of the extended dataset is evaluated in terms of average precision and recall metrics. Finally, Section 4 concludes this paper by summarizing the key findings, the contributions of the research, and potential directions for future work.

## 2. Materials and Methods

Facial image analysis datasets vary significantly in size, quality, and the specific tasks they support. Large-scale datasets like VGG-Face [71] and CelebA [72] have driven advancements in face recognition and attribute prediction, while AffectNet [73] and MORPH have enabled progress in specialized tasks such as emotion recognition and age estimation. However, many datasets still suffer from challenges like demographic bias, class imbalance, and insufficient diversity, which limits their generalizability and fairness. Addressing these limitations, future efforts should focus on creating datasets that encompass a broader spectrum of age, race, and environmental conditions to support the development of more equitable and robust facial analysis systems.

Despite the growing variety of datasets, very few focus on eyewear detection, an essential aspect of occlusion analysis in facial recognition. Currently CelebAMask-HQ [51] stands as the only large-scale, publicly available dataset that includes label information on glasses through segmentation masks. A key drawback of the CelebAMask-HQ dataset is its limited demographic diversity as it primarily contains celebrity images, which results in a narrower representation of age groups, ethnicities, and environmental conditions compared to more diverse datasets like FFHQ [29]. The FFHQ Glasses Detection Dataset presented in the current research fills this gap by providing bounding box annotations specifically for glasses, increasing the potential for accurate facial analysis in the presence of eyewear. This section summarizes the FFHQ Glasses Detection Dataset and highlights its importance compared to other well-known datasets.

### 2.1. The Need for Eyewear Detection Datasets and the Purpose of FFHQ Glasses Detection Extension

The dataset of annotated images of people wearing glasses has a number of research applications in various fields. The addition of eyewear labels to the FFHQ dataset adds value to the dataset and the image analysis research community. A non-exhaustive list of potential applications of such dataset could include the following:

- Development of eyeglasses detection algorithms to enhance facial recognition systems by reducing errors caused by occlusions and improving accuracy across various environmental conditions;
- Creation of segmentation prompts using foundation models to ensure precise separation of eyeglasses from facial features for more accurate detection;
- Implementation of virtual try-on systems for online eyewear retailers, allowing users to visualize different eyeglasses styles without visiting physical stores, improving user experience;
- Designing an algorithm for eyeglasses type recognition in natural environments, accounting for lighting variations and complex backgrounds;
- Enhancing face recognition models by improving robustness against variations introduced by eyeglasses, such as occlusions and reflections;
- Utilization of eyeglasses detection in augmented reality (AR) and virtual reality (VR) applications for accurate rendering of virtual content on user faces, improving interaction realism through better positioning and scaling;
- Integration of eyeglasses detection into driver monitoring systems to assess attention and distinguish between prescription glasses and sunglasses, contributing to enhanced road safety;
- Development of inpainting algorithms to reconstruct occluded facial regions caused by eyeglasses, improving the quality of facial images;
- Study of the impact of glare and reflections caused by eyeglasses along with research on removing these artifacts from images to enhance clarity in various applications;
- Enhancement of human–computer interaction (HCI) systems by adjusting screen settings based on the presence of eyeglasses, reducing glare and improving comfort and accessibility;
- Research on improving facial expression recognition in the presence of eyeglasses by minimizing the effects of occlusion on key facial features;
- Application of eyeglasses detection in security and surveillance systems, helping identify individuals attempting to disguise themselves and enhancing identification in high-security environments;
- Personalization of digital marketing by targeting advertisements based on the detection of eyeglasses, leading to more relevant product recommendations;
- Development of assistive technologies for visually impaired or elderly users, utilizing eyeglasses detection to issue alerts for misplaced glasses and monitor eye strain;
- Analysis of aesthetic appeal and fashion trends in eyewear by leveraging eyeglasses detection datasets, providing insights into consumer behavior and preferences;
- Benchmarking of eyeglasses detection algorithms across diverse cases, such as varying facial orientations, lighting conditions, and eyewear types;
- Enhancement of driver and pedestrian safety systems by detecting eyeglasses and adjusting vehicle systems to mitigate glare or to compensate for visual impairments;
- Application of eyeglasses detection in behavioral studies to explore the social and psychological impact of wearing glasses, such as effects on perceived trustworthiness and intelligence;
- Development of accessibility features on digital platforms, optimizing display settings for users wearing prescription glasses and improving usability;
- Research into pupil detection models in the presence of eyeglasses, accounting for occlusion and glare effects to improve accuracy in eye-tracking applications;
- Use of eyeglasses detection datasets in demographic studies, enabling analysis of eyewear prevalence across different populations and aiding surveys and research on vision correction trends.

### 2.2. Summary of the FFHQ Glasses Detection Dataset

#### 2.2.1. FFHQ Glasses Detection vs. CelebAMask-HQ Datasets

The CelebAMask-HQ and FFHQ (Flickr-Faces-HQ) datasets are both popular in machine learning, computer vision, and facial recognition research, but they have different characteristics in terms of structure, annotation, and diversity (Table 1).

CelebAMask-HQ is an extension of the CelebA-HQ dataset, itself derived from the original CelebA dataset, which primarily consists of celebrity images. CelebAMask-HQ offers around 30,000 high-resolution images (1024 × 1024 pixels) of celebrity faces, with its defining feature being pixel-wise semantic segmentation masks. These masks annotate 19 specific facial attributes, including components such as hair, eyes, nose, mouth, and accessories like glasses or hats. This level of detailed annotation makes CelebAMask-HQ particularly suitable for tasks requiring precise facial attribute manipulation, segmentation, landmark detection, face swapping, or facial feature editing [74]. The dataset’s primary application areas include face segmentation and image manipulation, where the accurate delineation of facial features is essential.

FFHQ was developed by NVIDIA and contains 70,000 high-resolution images (1024 × 1024) sourced from Flickr (San Francisco, CA, USA). The primary goal of FFHQ is to support the development of generative models, particularly GANs, such as StyleGAN [29]. Unlike CelebAMask-HQ, FFHQ does not include pixel-wise annotations or semantic segmentation masks, focusing instead on a broad range of diversity in terms of age, ethnicity, lighting, and background. FFHQ encompasses a wider array of facial features and environmental conditions, making it highly useful for training generative models or tasks that require generalization across diverse facial types and contexts. It was specifically designed to reduce biases present in facial datasets and is commonly used in applications like image synthesis, facial recognition, and other tasks where diversity in the dataset is critical. Extending the FFHQ dataset to include annotations for facial analysis would increase the value of the dataset.

The image quality of both datasets is equally high, with a resolution of 1024 × 1024 pixels. However, there is a significant difference in their scope of diversity. CelebAMask-HQ is limited to celebrity faces, meaning it lacks the broad range of facial features, age groups, and ethnicities found in FFHQ. This limitation makes CelebAMask-HQ more constrained as it tends to focus on well-known individuals, often leading to a narrower representation in terms of demographic variety. Although it still provides variation in gender and some ethnic diversity, it does not capture the same level of representation across different age groups or less familiar faces. FFHQ, by contrast, was explicitly designed to include a wide variety of facial appearances and environmental factors, making it more suitable for research requiring a comprehensive range of face types.

The availability of annotations is another key distinction between the two datasets. CelebAMask-HQ provides detailed pixel-wise segmentation masks, which are highly valuable for tasks involving facial feature manipulation or segmentation. The presence of 19 annotated facial components allows researchers to conduct fine-grained analysis and manipulation of individual facial attributes, offering a strong ground truth for tasks like facial parsing. FFHQ, in comparison, does not offer such detailed annotations. It focuses on image diversity for tasks like generative modeling, where pixel-wise labels are not necessarily required. Researchers working with FFHQ for tasks involving facial segmentation or detailed analysis often need to rely on external tools to generate the necessary labels or masks.

In terms of use cases, CelebAMask-HQ is primarily employed in tasks involving face segmentation, facial attribute manipulation, and image editing due to its rich segmentation annotations. Its application is well suited to research requiring precise control over individual facial attributes or structural components. On the other hand, FFHQ is commonly used for training generative models such as GANs due to its image diversity and focus on reducing bias. It is particularly well suited for applications involving facial recognition, image synthesis, and tasks requiring generalization across varied demographic groups.

The size of the datasets also differs considerably. FFHQ contains 70,000 images, offering a much larger dataset for training and testing models, whereas CelebAMask-HQ provides around 30,000 images. This difference in dataset size can impact the choice of dataset, depending on the research task at hand. While FFHQ’s larger dataset size is advantageous for models requiring extensive training data, CelebAMask-HQ’s segmentation data offers unique benefits for tasks involving detailed facial analysis.

Despite their strengths, both datasets have challenges. CelebAMask-HQ’s focus on celebrity faces introduces a bias that may limit its generalizability to non-celebrity populations. The narrower demographic representation can be a drawback for tasks requiring broader diversity. FFHQ, while offering greater diversity, lacks the detailed facial annotations necessary for tasks involving pixel-level analysis or manipulation, which may necessitate additional preprocessing.

#### 2.2.2. Features of FFHQ Glasses Detection Extension

**Figure 2:**
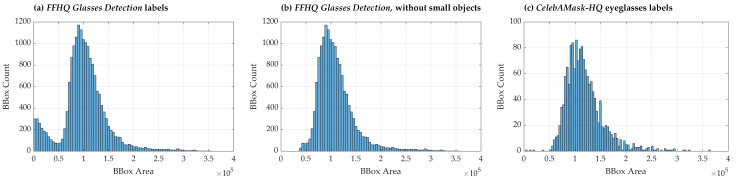
Distributions of the eyeglasses bounding box (BBox) area. Plots (**a**,**b**) show the distribution of eyeglasses BBox areas in the FFHQ dataset, while (**c**) represents the distribution for the CelebAMask-HQ dataset. Plot (**b**) differs from (**a**) as it illustrates the BBox area distribution for a subset of the FFHQ dataset used in baseline model experiments, excluding extremely small glasses objects. BBox areas were computed for 1024×1024 resolution images.

The distribution of eyeglasses bounding box (BBox) areas was analyzed for both the FFHQ and CelebAMask-HQ datasets (Figure 2). Three distinct plots were generated to visualize the area distribution of the eyeglasses BBoxes. The BBox area distribution for all detected eyeglasses in the FFHQ dataset is presented in a plot (Figure 2a), offering an overview of the full range of eyeglasses sizes. A subset of the FFHQ dataset used in baseline model experiments is shown in a plot (Figure 2b), where extremely small eyeglasses objects were excluded to distance them from the object size range (extremely small eyeglasses), which is where false positives are more likely. This filtering was applied to improve model robustness by removing objects that may have been inaccurately annotated or poorly detected. The distribution of the eyeglasses BBox areas in the CelebAMask-HQ dataset was illustrated in a plot (Figure 2c), allowing for a direct comparison with the FFHQ dataset. Differences between the two distributions revealed dataset-specific characteristics, including potential variations in annotation methods and the range of eyeglasses sizes represented.

**Figure 3:**
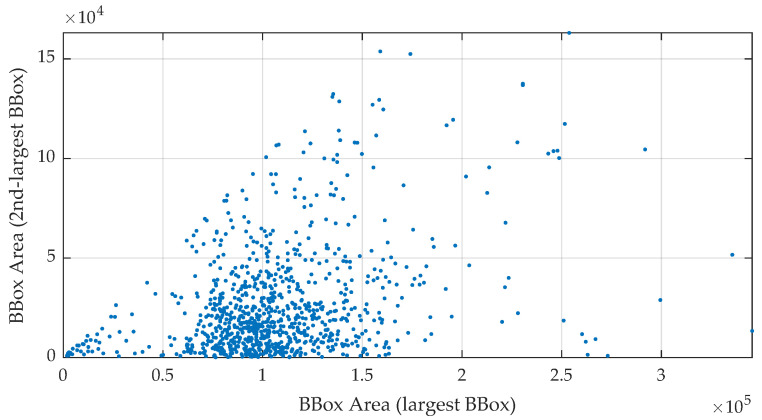
Co-occurrence plot of the largest and second-largest eyeglasses object areas in the same image. The plot visualizes the relationship between the areas of the largest and second-largest eyeglasses objects within images that contain more than one instance of eyeglasses. This provides insights into the relative sizes of multiple glasses in the same image, offering a deeper understanding of the distribution of the object sizes in such cases.

A co-occurrence plot (Figure 3) was also generated to examine the relationship between the areas of the largest and second-largest eyeglasses objects within the same image. This analysis was limited to images containing more than one instance of eyeglasses. The plot visualizes the size relationship between these objects, providing insights into the variability of multiple eyeglasses within individual images. This information is important for debugging object detection models as the presence of multiple eyeglasses with different sizes can introduce complexities in detection tasks. The co-occurrence plot, therefore, provides valuable data for developing models that can effectively handle cases of occlusion or overlapping objects.

#### 2.2.3. Bridging FFHQ Extensions: Glasses Detection, Aging, and Features Datasets

**Figure 4:**
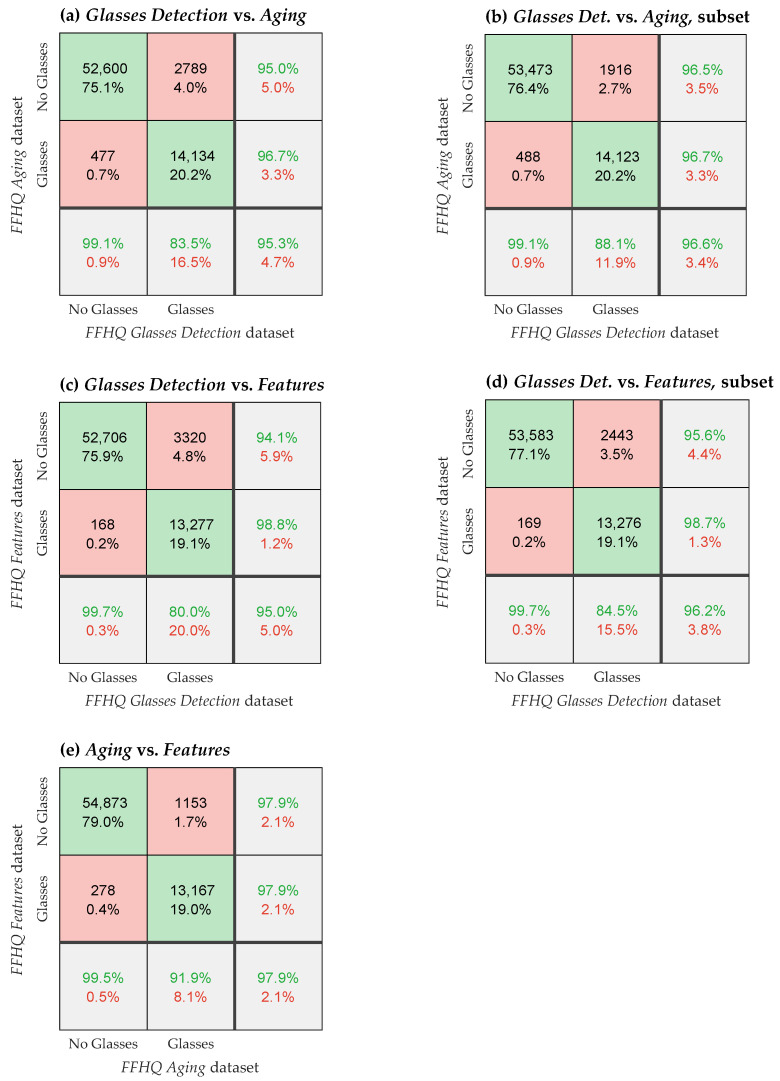
Confusion matrices showing the discrepancies in glasses attributes between the FFHQ dataset extensions. The figure presents confusion matrices that compare the glasses attribute (glasses or no glasses) across three FFHQ dataset extensions: Glasses Detection, Aging, and Features. Plots (**a**,**b**) compare Glasses Detection with Aging, while (**c**,**d**) compare Glasses Detection with Features. Plot (**e**) compares Aging with Features. In plots (**b**,**d**), the Glasses Detection subset without very small glasses objects is used as the Aging and Features datasets typically label glasses on the central face in the image. For comparisons involving the Features dataset, only instances with available Features labels are included. The green font or background represents correct results, the red font or background represents incorrect results.

**Figure 5:**
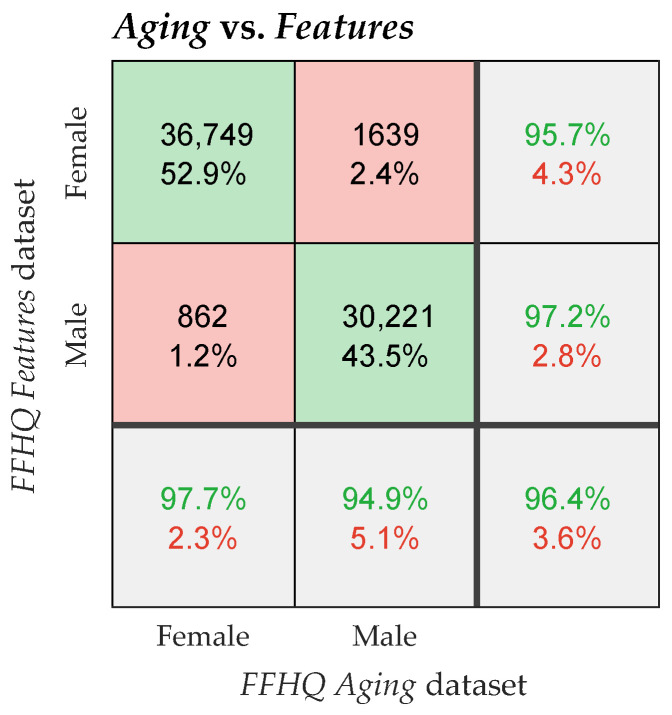
Confusion matrix showing the gender attribute discrepancies between the Aging and Features FFHQ dataset extensions. The confusion matrix illustrates the discrepancies in gender labeling (female or male) between the Aging and Features extensions, considering only images that have glasses labels in the Glasses Detection extension. The green font or background represents correct results, the red font or background represents incorrect results.

**Figure 6:**
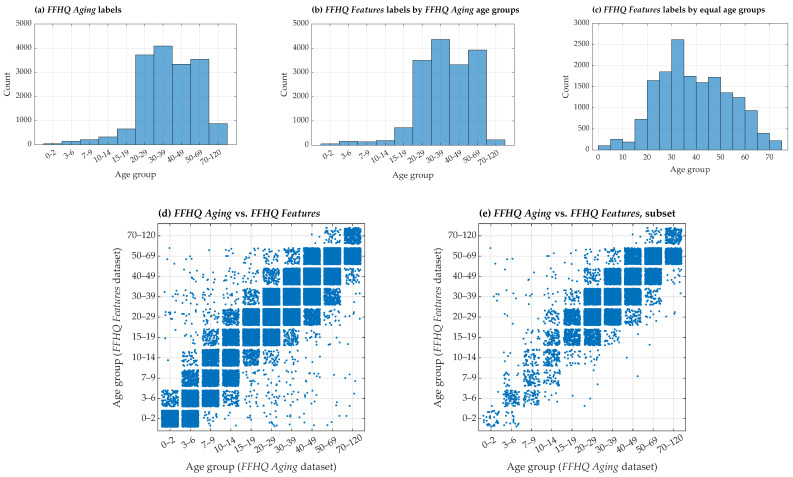
Age distributions in the Aging and Features extensions of the FFHQ dataset. Plots present the age distributions across the Aging and Features extensions, considering only images with glasses labels from the Glasses Detection extension (except for (**d**)): (**a**) shows the age distribution in the Aging extension, with labels grouped according to the original intervals in the dataset; (**b**) presents the age distribution in the Features extension, with labels grouped similarly to those in the Aging extension; (**c**) displays the age distribution in the Features extension with labels grouped into equal intervals; and (**d**,**e**) are scatter plots comparing ages between the Aging and Features datasets, with (**d**) including all images and (**e**) only focusing on images where glasses are present in the Glasses Detection extension (age binning was based on groups provided by the Aging dataset with noise added to the bin IDs to prevent instance overlap in plots).

**Figure 7:**
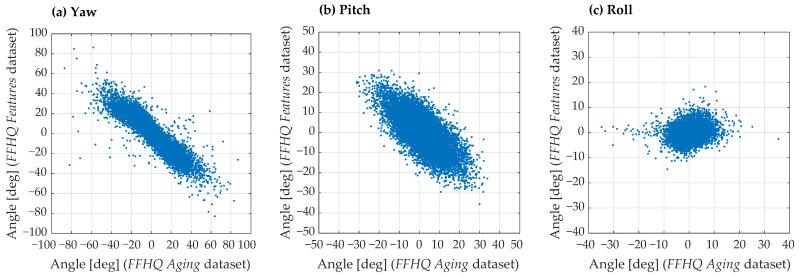
Scatter plots comparing the head pose values—yaw (**a**), pitch (**b**), and roll (**c**)—between the Aging and Features extensions of the FFHQ dataset. Only images with glasses labels from the Glasses Detection extension were included in this analysis. The plots provide insights into the alignment and discrepancies in the head pose annotations across the two dataset extensions.

The FFHQ Glasses Detection dataset extends the FFHQ dataset by introducing bounding box (BBox) labels for eyewear detection, complementing existing extensions like FFHQ Aging (https://github.com/royorel/FFHQ-Aging-Dataset (accessed on 21 August 2024)) [75] and FFHQ Features datasets (https://github.com/DCGM/ffhq-features-dataset (accessed on 21 August 2024)). By focusing on the localization of glasses, it enhances facial analysis. Unlike FFHQ Aging and Features, which provide probabilistic labels such as facial attributes, FFHQ Glasses Detection offers precise localization. Together these datasets expand the scope of facial image analysis by integrating various facial attributes like aging, gender, and head pose and eyewear detection. Their combined usage allows for more comprehensive research across domains such as computer vision, facial recognition, and human–computer interaction, enriching the utility of FFHQ-based models. Therefore, the summary plots provide valuable insights into the datasets (Figure 4, Figure 5, Figure 6 and Figure 7).

The FFHQ Aging dataset [75] introduces age labels for FFHQ images, enabling research in facial aging and progression. By extending FFHQ’s demographic diversity, this dataset supports studies on how facial features evolve with age. While age is an important factor in many facial analyses, the presence of eyeglasses can significantly affect visual perception and model accuracy, particularly in age progression tasks. The integration of glasses detection provides a complementary aspect to FFHQ-Aging by allowing age-aware models to account for eyewear’s impact on facial structure. The Aging dataset provides the following information for each image: glasses type, age, gender, head pose, and eye occlusion score.

The FFHQ Features dataset expands FFHQ by providing metadata related to various facial features such as the presence of glasses, gender, age, head pose, mustache/beard/sideburns probability, emotion, blur level, exposure, noise level, eye makeup and lip makeup, accessories, occlusion, hair, smile probability, and face bounding rectangle.

The Aging and Features datasets deliver probabilistic scores for glasses detection but lack the precise localization of eyewear. FFHQ Glasses Detection addresses this gap by offering detailed BBox annotations, making it possible to not only detect the presence of glasses, but also to pinpoint their exact position in images. These labels allow for improved downstream tasks such as facial attribute extraction and occlusion handling, enhancing the capabilities initiated by the FFHQ Features extension.

The integration of these datasets offers advantages for facial analysis tasks; however, achieving high-quality results requires careful analysis of their consistency and discrepancies. The summary plots presented in this section provide valuable insights into the consistency and alignment between the three FFHQ extensions.

The confusion matrices in Figure 4 illustrate the relationships between glasses detection labels (presence or absence of glasses) across the three FFHQ dataset extensions. Each sub-plot provides insights into how well the datasets agree on the labeling of glasses. In the Glasses Detection dataset, the glasses attribute was determined based on the presence of bounding boxes in the images.

The gender confusion matrix (Figure 5) highlights the gender labeling discrepancies between the FFHQ Aging and FFHQ Features datasets, focusing on images where glasses are present in the Glasses Detection extension. Given that glasses may obscure some facial features, gender misclassification is more likely to occur in images with glasses.

The age distributions (Figure 6) illustrate the distribution of ages across the FFHQ Aging and FFHQ Features datasets for images that have glasses labels in the FFHQ Glasses Detection dataset.

The scatter plots (Figure 7) compare the head pose values (yaw, pitch, and roll) between the FFHQ Aging and FFHQ Features datasets for images with glasses. The presence of glasses can introduce minor errors, particularly in extreme head poses where parts of the face may be occluded.

### 2.3. Protocol of FFHQ Image Annotation for Eyewear (Glasses) Detection

The process of labeling eyeglasses in the image dataset followed a semi-automatic approach, combining zero-shot object detection models with manual verification. Initially, the images were annotated using three zero-shot object detection foundation models: DETIC [76], GroundingDINO [77], and OWLv2 [78]. These models were deployed through the Roboflow Autodistill tool [79], which enabled the automatic generation of labels for eyeglasses within the dataset.

All three models share a basis in transformers but with variations in how they handle supervision. DETIC and GroundingDINO are based on DETR-like architectures and OWLv2 uses more CLIP-like contrastive mechanisms. DETIC integrates image–text matching for object detection, enabling the identification of objects based on textual descriptions [76]. Its zero-shot nature allows it to generalize beyond its training set, detecting eyeglasses even in novel images where conventional models may struggle. GroundingDINO leverages vision-language alignment, allowing it to detect objects described by text prompts without requiring object-specific training [77]. This model provides robust localization capabilities for a wide variety of objects, making it well suited for identifying eyeglasses in diverse image contexts. OWLv2 is another zero-shot detection model that utilizes a vision-language transformer architecture [78]. It can detect objects by associating textual labels with visual features, offering high versatility in object detection, particularly for objects such as eyeglasses, which can vary significantly in shape, size, and style across the dataset.

Following the initial detection phase, where bounding boxes (BBoxes) were generated using DETIC, GroundingDINO, and OWLv2, a structured review strategy was applied to further refine the labels. The performance of the models varied, with DETIC returning the fewest labels but maintaining a low false-positive rate, while GroundingDINO returned a larger number of BBoxes with a higher false-positive rate. OWLv2 performed similarly to DETIC but was able to detect smaller objects, resulting in slightly more detections overall. Based on these observations, the dataset was divided into review chunks that required different levels of manual correction.

**Figure 8:**
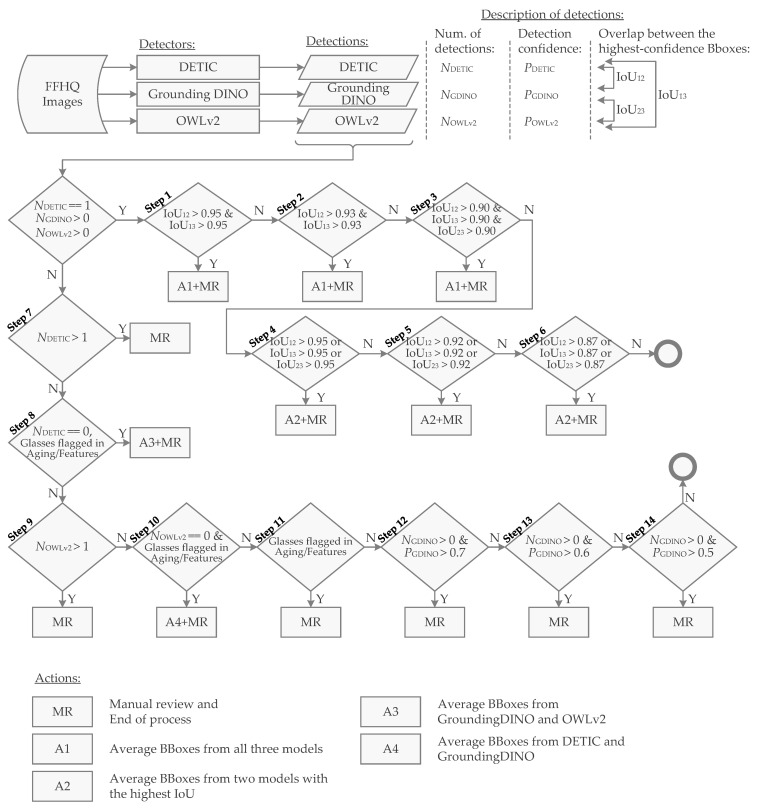
Flowchart of the semi-manual labeling protocol for eyeglasses detection. The protocol iteratively refines bounding box (BBox) annotations by integrating outputs from multiple detection models, including DETIC, GroundingDINO, and OWLv2, with additional dataset features. The process ensures a systematic progression from simpler cases requiring minimal manual correction to more complex cases necessitating greater human intervention. Iterative refinement incorporates model consensus and additional data sources, and it is supported by thresholds designed to select a subset of images for manual review at each step.

The semi-manual labeling protocol for detecting eyeglasses was designed to iteratively refine bounding box (BBox) annotations by utilizing the outputs of multiple detection models and additional dataset features. The following steps describe the iterative review strategy, starting with the simplest cases requiring minimal manual correction and progressing to more complex cases that demand greater attention (Figure 8).

Input data:

- Detections from GroundingDINO, DETIC, and OWLv2 models, providing bounding boxes (BBoxes) for eyeglasses.
- Additional features regarding the existence of eyeglasses derived from FFHQ Aging and FFHQ Features datasets.

Notation:

- $N_{\mathrm{DETIC}}$—the number of eyeglass detections from DETIC.
- $N_{\mathrm{GDINO}}$—the number of eyeglass detections from GroundingDINO.
- $N_{\mathrm{OWLv}2}$—the number of eyeglass detections from OWLv2.
- ${\mathrm{IoU}}_{12}$—the Intersection over Union (IoU) between the highest-confidence BBoxes from DETIC and GroundingDINO.
- ${\mathrm{IoU}}_{13}$—the IoU between the highest-confidence BBoxes from DETIC and OWLv2.
- ${\mathrm{IoU}}_{23}$—the IoU between the highest-confidence BBoxes from GroundingDINO and OWLv2.

Manual review procedure:

**Step 1:**  High Agreement, Single BBox from DETIC
  - Condition: $N_{\mathrm{DETIC}}==1$, $N_{\mathrm{GDINO}}>0$, and $N_{\mathrm{OWLv}2}>0$.
  - IoU Thresholds: ${\mathrm{IoU}}_{12}\geq 0.95$ and ${\mathrm{IoU}}_{13}\geq 0.95$.
  - Action: Generate an initial BBox by averaging the highest-confidence BBoxes from all three models. Review this BBox manually.
**Step 2:**  Slightly Lower IoU Threshold
  - Condition: Same as Step 1.
  - IoU Thresholds: ${\mathrm{IoU}}_{12}\geq 0.93$ and ${\mathrm{IoU}}_{13}\geq 0.93$.
  - Action: Follow the same procedure as Step 1.
**Step 3:**  Lower IoU with GroundingDINO and OWLv2
  - Condition: Same as Step 1.
  - IoU Thresholds: ${\mathrm{IoU}}_{12}\geq 0.90$, ${\mathrm{IoU}}_{13}\geq 0.90$, and ${\mathrm{IoU}}_{23}\geq 0.90$.
  - Action: Follow the same procedure as Step 1.
**Step 4:**  High IoU Between Any Two Models
  - Condition: Same as Step 1.
  - IoU Thresholds: ${\mathrm{IoU}}_{12}\geq 0.95$ or ${\mathrm{IoU}}_{13}\geq 0.95$, or ${\mathrm{IoU}}_{23}\geq 0.95$.
  - Action: Generate an initial BBox by averaging the highest-confidence BBoxes from two models with the highest IoU. Review the BBox.
**Step 5:**  Moderate IoU Agreement Between Two Models
  - Condition: Same as Step 1.
  - IoU Thresholds: ${\mathrm{IoU}}_{12}\geq 0.92$ or ${\mathrm{IoU}}_{13}\geq 0.92$, or ${\mathrm{IoU}}_{23}\geq 0.92$.
  - Action: Follow the same procedure as Step 4.
**Step 6:**  Lower IoU Agreement Between Two Models
  - Condition: Same as Step 1.
  - IoU Thresholds: ${\mathrm{IoU}}_{12}\geq 0.87$ or ${\mathrm{IoU}}_{13}\geq 0.87$, or ${\mathrm{IoU}}_{23}\geq 0.87$.
  - Action: Follow the same procedure as Step 4.
**Step 7:**  Multiple Detections from DETIC
  - Condition: $N_{\mathrm{DETIC}}>1$.
  - Action: Manually review all detections, comparing with other models to confirm the most accurate BBoxes.
**Step 8:**  No Detection from DETIC, Eyeglasses Present in FFHQ
  - Condition: $N_{\mathrm{DETIC}}==0$, but eyeglasses are flagged in FFHQ Aging or FFHQ Features datasets.
  - Action: Generate an initial BBox by averaging the highest-confidence BBoxes from GroundingDINO and OWLv2. Review the BBox.
**Step 9:**  Multiple Detections from OWLv2
  - Condition: $N_{\mathrm{OWLv}2}>1$.
  - Action: Manually review all detections, adjusting BBoxes as necessary.
**Step 10:**  No Detection from OWLv2, Eyeglasses Present in FFHQ
  - Condition: $N_{\mathrm{OWLv}2}==0$, but eyeglasses are flagged in FFHQ Aging or FFHQ Features datasets.
  - Action: Generate an initial BBox by averaging the highest-confidence BBoxes from DETIC and GroundingDINO. Review the BBox.
**Step 11:**  Positive Eyeglasses Feature in FFHQ
  - Condition: FFHQ datasets indicate the presence of eyeglasses.
  - Action: Manually review the image for potential eyeglass detection, creating or correcting BBoxes as necessary.
**Step 12:**  GroundingDINO Detection with High Confidence
  - Condition: $N_{\mathrm{GDINO}}>0$ and the highest-confidence BBox from GroundingDINO has a confidence score >0.7.
  - Action: Manually review and adjust BBox as necessary.
**Step 13:**  GroundingDINO Detection with Moderate Confidence
  - Condition: $N_{\mathrm{GDINO}}>0$ and the highest-confidence BBox from GroundingDINO has a confidence score >0.6.
  - Action: Follow the same procedure as Step 12.
**Step 14:**  GroundingDINO Detection with Low Confidence
  - Condition: $N_{\mathrm{GDINO}}>0$ and the highest-confidence BBox from GroundingDINO has a confidence score >0.5.
  - Action: Follow the same procedure as Step 12.

The protocol ensures a systematic progression from simpler cases requiring minimal manual correction to more complex cases that necessitate greater human intervention. Each step incorporates iterative refinement of bounding boxes using model consensus and additional data sources.

### 2.4. Baseline Eyewear Detection Results for Dataset Evaluation

The evaluation of the newly created FFHQ Glasses Detection dataset was an important aspect of this research. The goal of the evaluation was to determine whether using this new dataset provided any advantages over existing alternatives, such as the CelebAMask-HQ dataset. To allow for a robust comparison, a cross-dataset validation approach was adopted. This method evaluates the generalizability of models trained on one dataset when applied to a different dataset, thus testing their robustness in real-world scenarios where training and testing data distributions may differ.

The evaluation procedure involved three datasets: FFHQ Glasses Detection, CelebAMask-HQ, and a Mixed Dataset (which was created by merging both datasets). The evaluation followed a protocol similar to three-fold cross-validation, which was extended to accommodate testing on additional datasets. Each dataset was randomly partitioned into three folds. For each dataset, three separate training runs were performed, with one fold held out for evaluation while the remaining two folds were used for training. After training, models were tested on the held-out fold of the training dataset and on the corresponding fold of the other datasets to assess cross-dataset performance.

The evaluation metrics used were Average Precision (AP) and Average Recall (AR), which was calculated for each dataset. These metrics were implemented using KerasCV’s COCO evaluation framework [7,80]. AP and AR metrics following the COCO implementation were calculated as averages across multiple Intersection over Union (IoU) thresholds. Specifically, 10 IoU thresholds, ranging from 0.50 to 0.95 in steps of 0.05, were used for evaluation.

**Figure 9:**
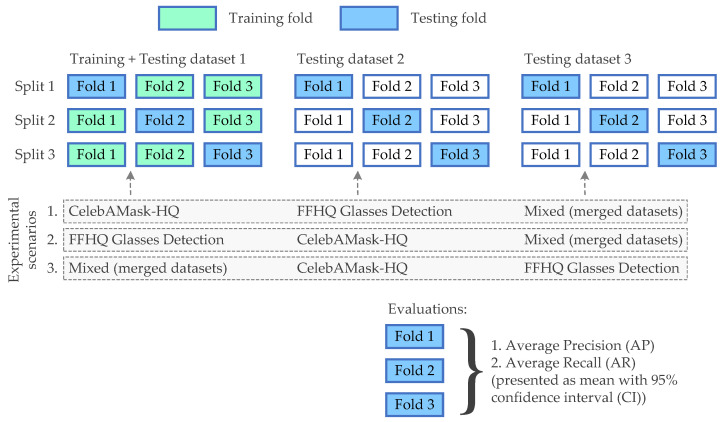
Overall block diagram of the experiment.

After three-fold cross-validation, the results were aggregated over the three runs, and the final scores were reported as mean values along with 95% confidence intervals to provide a measure of statistical significance and robustness. The cross-dataset validation setup, which is illustrated in Figure 9, helped to assess model performance in conditions where the data distributions differ, simulating real-world deployment scenarios.

For the evaluation, the Keras RetinaNet model (https://keras.io/api/keras_cv/models/tasks/retinanet/ (accessed on 21 August 2024)) was employed using four different backbone models: YOLOv8xs, YOLOv8s, YOLOv8m [81], and MobileNetV3s [82]. RetinaNet is well suited for object detection tasks as it incorporates a feature pyramid network (FPN) for efficient multi-scale detection [83]. Additionally, it uses the Focal Loss function, which addresses the class imbalance problem by assigning greater importance to harder-to-classify examples. The models were initialized with pre-trained weights from COCO or ImageNet datasets, ensuring that the transfer learning approach could be leveraged for improved initial performance.

Key characteristics of the model backbones, such as the number of parameters, network depth, input resolution, and latency on both CPU and GPU, are provided in Table 2. These factors were taken into consideration when selecting models for evaluation, balancing model complexity against computational efficiency.

The training process was empirically configured to achieve a balance between the computational cost and model performance, as indicated by the convergence trends observed in the initial experiments. The models were trained for 150 epochs, with 500 steps per epoch and a batch size of 64. The Adam optimizer was employed, with a learning rate that was linearly decreased from $5\times 10^{-4}$ to $1\times 10^{-4}$ throughout training. The input image resolution for all models was set to 512×512 pixels.

To enhance model generalization, an augmentation pipeline was applied during training using the Albumentations library. This pipeline included the following transformations: (a) Blur; (b) one of ChannelShuffle or ColorJitter; (c) one of Sharpen, RandomBrightnessContrast, MotionBlur, or Blur; (d) ShiftScaleRotate; (e) HorizontalFlip; and (f) CoarseDropout.

The loss function for the training RetinaNet model consisted of two components: Focal Loss for classification and Smooth L1 Loss for bounding box regression.

### 2.5. Software Used

The software tools and programming languages used in this research were as follows:

- Python (version 3.11.9) (https://www.python.org, (accessed on 21 August 2024)) [84], an interpreted, high-level, and general-purpose programming language. Used for the machine learning applications.
- TensorFlow (version 2.17.0) with Keras (version 3.5.0) and KerasCV (version 0.9.0) (https://www.tensorflow.org (accessed on 21 August 2024)) [80,85,86], an open-source platform for machine learning. Used for the semi-automated eyewear labeling and for the training/testing of the baseline deep learning models.
- Albumentations (version 1.4.14) (https://albumentations.ai (accessed on 21 August 2024)) [87], a Python library for fast and flexible image augmentation. Used for the image augmentations during deep learning model training.
- OpenCV (version 4.10.0) (https://opencv.org/ (accessed on 21 August 2024)) [88], an open-source computer vision library. Used for image input/output and manipulations.
- Roboflow’s Autodistill tool (https://github.com/autodistill/autodistill (accessed on 21 August 2024)) and its foundation models for object detection. Tool used to generate initial annotations for eyewear detection on unannotated images in the FFHQ dataset.
- labelme (version 5.2.1) (https://github.com/wkentaro/labelme (accessed on 21 August 2024)) [89], a graphical image annotation tool. Used to review eyewear labels.

## 3. Results and Discussion

The performance of the baseline eyewear detection models was evaluated across three datasets: the created extention FFHQ Glasses Detection; the CelebAMask-HQ; and a mixed dataset, which combined both. Four models were trained using the RetinaNet framework, which incorporates four backbone architectures (YOLOv8xs, YOLOv8s, YOLOv8m, and MobileNetV3s). The following evaluation metrics were calculated using a cross-dataset validation approach: Average Precision (AP) and Average Recall (AR). These key results of the baseline experiments are summarized in Table 3 and Table 4. Additional detailed results are presented in Appendix A Table A1, Table A2, Table A3, Table A4, Table A5, Table A6, Table A7 and Table A8: Average Precision at IoU = 0.50; Average Precision at IoU = 0.75; Average Precision for small objects (area < $300^{2}$); Average Precision for medium objects ($300^{2}$ < area < $340^{2}$); Average Precision for large objects (area > $340^{2}$); Average Recall for small objects (area < $300^{2}$); Average Recall for medium objects ($300^{2}$ < area < $340^{2}$); and Average Recall for large objects (area > $340^{2}$). The thresholds for small, medium, and large objects were determined based on the size distribution of the eyeglasses BBoxes, where the aim was that the dataset was divided into equal subsets.

The Average Precision (AP) scores reflect the accuracy with which each model detected glasses across different datasets. When trained on FFHQ Glasses Detection, all of the YOLOv8-based models demonstrated improved AP scores by achieving AP values of 0.88 on the FFHQ dataset. MobileNetV3s showed slightly lower AP values (0.83).

When applied to the CelebAMask-HQ dataset, the AP scores were slightly lower, which can be attributed to the more limited diversity in the CelebAMask-HQ’s annotations and its focus on celebrity images. Despite this, the models trained on the mixed dataset (a combination of both CelebAMask-HQ and FFHQ) consistently demonstrated strong performance across all test datasets, with AP scores close to 0.86 across the board.

The Average Recall (AR) scores, which reflect the completeness of the glasses detection, showed similar trends to the AP results. Models trained on FFHQ Glasses Detection and the mixed dataset consistently produced higher recall scores, with YOLOv8s and YOLOv8m leading with AR values close to 0.91 when tested on the FFHQ dataset. Slightly lower AR values were observed on the CelebAMask-HQ dataset, particularly for MobileNetV3s (AR = 0.80).

The YOLOv8 models consistently outperformed MobileNetV3s across datasets, with particularly strong results when trained on the FFHQ Glasses Detection dataset. YOLOv8m and YOLOv8s exhibited similarly robust performance, with marginal improvements in AP and AR over YOLOv8xs in some instances.

The consistent performance of the models trained on the mixed dataset suggests that combining datasets with different characteristics allows the models to generalize better, improving both precision and recall across diverse facial images and variations in glasses appearance.

A key aspect of this study was the cross-dataset validation, where models trained on one dataset were tested on another to assess their robustness and generalizability. Models trained on the extended FFHQ dataset outperformed those trained on CelebAMask-HQ when applied to both CelebAMask-HQ and FFHQ. The YOLOv8-based models demonstrated the highest levels of generalization, with only a minor drop in performance when tested on datasets they had not been trained on. This suggests that the diversity of the FFHQ dataset, particularly with respect to age, ethnicity, and lighting conditions, makes it highly effective for training glasses detection models that generalize well to other datasets.

Furthermore, the mixed dataset produced slightly more balanced results across both datasets, indicating that combining diverse datasets can enhance the robustness of the models. This result is particularly relevant for real-world applications where training data might differ from the data encountered during deployment.

One of the main contributions of this work is the inclusion of diverse glasses annotations in the FFHQ dataset. The evaluation results show that models trained on the extended FFHQ dataset performed better than those trained on the smaller, less diverse CelebAMask-HQ dataset. The higher performance on FFHQ reflects the impact of diversity in eyewear types, sizes, and styles, which was not adequately represented in the CelebAMask-HQ dataset. The larger size of the FFHQ dataset allowed for better coverage of edge cases, such as variations in transparency, reflections, and lighting conditions. These factors often degrade the performance of traditional detection models but were effectively handled by models trained on the extended FFHQ dataset.

Despite the success of the baseline models, several challenges remain. Eyewear detection is still sensitive to occlusion and glare, which can significantly reduce the detection accuracy in certain conditions. While data augmentation techniques were employed to mitigate these issues, the impact of extreme lighting and reflections remains a concern, particularly in images with complex backgrounds.

Future research could explore the use of more advanced architectures or ensemble methods, which could combine the strengths of multiple models. Additionally, the integration of pixel-wise annotations, as seen in CelebAMask-HQ, could provide more granular control over glasses localization, enabling more accurate detections in challenging conditions.

Another promising research direction is the development of specialized models that can handle specific challenges, such as transparent or reflective glasses. By leveraging the strengths of the extended FFHQ dataset, researchers can continue to improve detection algorithms for a wide range of applications, from augmented reality to facial recognition and assistive technologies.

## 4. Conclusions

This research addresses the existing gap in publicly available datasets for eyeglasses detection by extending the FFHQ dataset with detailed bounding box annotations. The newly created FFHQ Glasses Detection dataset, an extension to the FFHQ dataset, offers significant improvements over CelebAMask-HQ in terms of size and diversity, providing a more robust resource for training and testing machine learning models. Baseline experiments with YOLOv8 and MobileNetV3 demonstrate that models trained on this extended dataset achieve higher performance, both in precision and recall, compared to those trained on smaller and less diverse dataset CelebAMask-HQ. Cross-dataset validation results show that the diversity inherent in the extended FFHQ dataset leads to better generalization in real-world scenarios, highlighting the dataset’s utility for a range of applications, including facial recognition, augmented reality, and assistive technologies.

Eyewear detection remains challenging due to factors such as occlusion, glare, and lighting variations. These issues, while partially mitigated through data augmentation techniques, suggest directions for further research. Future work could explore more advanced model architectures, pixel-wise annotations, or specialized models capable of handling the specific challenges posed by transparent and reflective glasses. The release of this extended FFHQ dataset is expected to inspire further advances in eyewear detection, offering a new benchmark for researchers and practitioners in the field of facial analysis.

## Figures and Tables

**Table 1:** Comparative summary of the FFHQ and CelebAMask-HQ datasets.

Feature	CelebAMask-HQ	FFHQ (Flickr-Faces-HQ)
Origin	Derived from CelebA-HQ and based on celebrity images	Created by NVIDIA and sourced from Flickr
Number of images	30,000	70,000
Number of images containing eyewear	1349	16,923 (labels are provided in this research)
Resolution	1024 × 1024	1024 × 1024
Diversity	Moderate diversity (predominantly celebrities and less diverse in age and backgrounds)	High diversity (age, ethnicity, backgrounds, and lighting)
Annotations	Pixel-wise segmentation masks for 19 facial components (hair, eyes, nose, mouth, etc.)	No pixel-wise annotations
Main purpose	Facial attribute manipulation, semantic segmentation, and facial landmark detection	Generative modeling (e.g., GAN training), face synthesis, and facial recognition
Facial component details	Detailed pixel-wise facial segmentation for 19 facial attributes	No explicit facial component annotations
Age representation	Primarily adults (celebrities)	Wide range (including children, adults, and the elderly)
Ethnic and cultural diversity	Moderate due to celebrity focus	High
Image backgrounds	More controlled, focused on clear images of celebrity faces	Varied (includes different lighting conditions, settings, etc.)
Applications	Facial attribute editing, face parsing, landmark detection, and face-swapping	Image synthesis, GANs, facial recognition, and transfer learning
Bias	Celebrity bias, and fewer diverse age and appearance groups	Designed to minimize bias through diversity
Challenges	Smaller dataset size and less diversity	Lacks detailed annotations for facial components

**Table 2:** Summary of the backbones.

Backbone	Parameters ^1^	Depth ^2^	Size (MB) ^3^	Input ^4^	Latency (ms) CPU ^5^	Latency (ms) GPU ^5^
YOLOv8xs COCO ^6^	1.28 M	27	4.87	$512^{2}$	484	24.8
YOLOv8s COCO ^6^	5.09 M	27	19.42	$512^{2}$	1070	33.2
YOLOv8m COCO ^6^	11.87 M	39	45.29	$512^{2}$	2210	44.9
MobileNetV3s ImageNet ^6^	939.12 K	34	3.58	$512^{2}$	479	29.3

^1^ The number of backbone’s parameters; ^2^ the number of convolutional layers; ^3^ the size of the weights file; ^4^ input size (resolution); ^5^ the time per inference step on CPU/GPU, which was evaluated by averaging 30 batches of size 32 and 10 repetitions (CPU: Intel Core i7 12,700K Processor; RAM: 128 GB; GPU: NVIDIA GeForce RTX 4090, 24 GB; platform: Windows Subsystem for Linux (WSL) 2); and ^6^ the backbones pretrained on the COCO or ImageNet datasets.

**Table 3:** Average Precision (AP) baseline results for eyeglasses detection on CelebAMask-HQ; FFHQ Glasses Detection; and on a mixed dataset, which combines both. Baseline models were trained on training folds of different datasets (CelebAMask-HQ, FFHQ Glasses Detection, and mixed) and were tested on all the remaining folds of each dataset. The results are presented as the means of three-fold cross-validation with 95% confidence intervals (CIs).

Model	Training Data	Test Data					
		CelebAMask-HQ		FFHQ Glasses Det.		Mixed (All)	
		Mean	95% CI	Mean	95% CI	Mean	95% CI
YOLOv8xs	CelebAMask-HQ	0.78	0.74–0.81	0.78	0.75–0.81	0.78	0.76–0.80
	FFHQ Glasses Det.	0.81	0.77–0.86	0.86	0.85–0.87	0.85	0.85–0.86
	Mixed (All)	0.81	0.75–0.87	0.86	0.85–0.87	0.85	0.85–0.86
YOLOv8s	CelebAMask-HQ	0.78	0.73–0.83	0.79	0.78–0.80	0.79	0.78–0.79
	FFHQ Glasses Det.	0.82	0.76–0.88	0.87	0.86–0.88	0.86	0.86–0.87
	Mixed (All)	0.82	0.76–0.88	0.87	0.85–0.88	0.86	0.85–0.87
YOLOv8m	CelebAMask-HQ	0.79	0.74–0.84	0.79	0.78–0.80	0.79	0.78–0.79
	FFHQ Glasses Det.	0.82	0.76–0.89	0.88	0.87–0.88	0.87	0.87–0.88
	Mixed (All)	0.82	0.79–0.86	0.86	0.84–0.89	0.86	0.84–0.88
MobileNetV3s	CelebAMask-HQ	0.75	0.70–0.81	0.75	0.73–0.77	0.75	0.73–0.77
	FFHQ Glasses Det.	0.79	0.76–0.82	0.83	0.81–0.85	0.83	0.82–0.84
	Mixed (All)	0.80	0.75–0.85	0.83	0.81–0.84	0.82	0.81–0.83

The YOLOv8 backbones were pretrained on the COCO dataset, and MobileNetV3 was pretrained on the ImageNet dataset.

**Table 4:** The Average Recall (AR) baseline results for eyeglasses detection on CelebAMask-HQ; FFHQ Glasses Detection; and a mixed dataset, which combines both. The baseline models were trained on training folds of different datasets (CelebAMask-HQ, FFHQ Glasses Detection, and mixed) and were tested on all the remaining folds of each dataset. The results are presented as the means of three-fold cross-validation with 95% confidence intervals (CIs).

Model	Training Data	Test Data					
		CelebAMask-HQ		FFHQ Glasses Det.		Mixed (All)	
		Mean	95% CI	Mean	95% CI	Mean	95% CI
YOLOv8xs	CelebAMask-HQ	0.83	0.80–0.87	0.83	0.82–0.85	0.83	0.82–0.85
	FFHQ Glasses Det.	0.86	0.82–0.89	0.89	0.89–0.90	0.89	0.89–0.89
	Mixed (All)	0.86	0.81–0.90	0.89	0.88–0.90	0.89	0.88–0.89
YOLOv8s	CelebAMask-HQ	0.84	0.80–0.88	0.84	0.84–0.84	0.84	0.83–0.84
	FFHQ Glasses Det.	0.86	0.81–0.91	0.90	0.89–0.91	0.90	0.89–0.91
	Mixed (All)	0.87	0.83–0.90	0.90	0.89–0.91	0.90	0.89–0.90
YOLOv8m	CelebAMask-HQ	0.85	0.81–0.88	0.84	0.83–0.85	0.84	0.84–0.85
	FFHQ Glasses Det.	0.87	0.82–0.92	0.91	0.90–0.91	0.90	0.89–0.91
	Mixed (All)	0.87	0.84–0.89	0.90	0.88–0.92	0.90	0.88–0.91
MobileNetV3s	CelebAMask-HQ	0.81	0.76–0.86	0.80	0.78–0.82	0.80	0.79–0.81
	FFHQ Glasses Det.	0.84	0.80–0.87	0.87	0.86–0.88	0.87	0.86–0.87
	Mixed (All)	0.84	0.81–0.87	0.86	0.84–0.88	0.86	0.85–0.88

The YOLOv8 backbones were pretrained on the COCO dataset, and MobileNetV3 was pretrained on the ImageNet dataset.

## Data Availability

The created extension of the FFHQ dataset for the eyewear detection is openly available and can be found at Zenodo (https://doi.org/10.5281/zenodo.14252074) (accessed on 21 August 2024).

## Abbreviations

The following abbreviations are used in this manuscript:

FFHQ	Flickr-Faces-HQ
CV	Computer vision
ML	Machine learning
DL	Deep learning
AI	Artificial intelligence
CNN	Convolutional neural network
ANN	Artificial neural network
DNN	Deep neural network
LR	Learning rate
BBox	Bounding box
AP	Average precision
AR	Average recall
CI	Confidence interval

## Appendix A

**Table A1:** Average Precision at the IoU = 0.50 (AP^IoU = 0.50^) baseline results for eyeglasses detection on CelebAMask-HQ; FFHQ Glasses Detection; and a mixed dataset, which combines both. Baseline models were trained on the training folds of different datasets (CelebAMask-HQ, FFHQ Glasses Detection, and mixed) and were tested on all the remaining folds of each dataset. The results are presented as the means of three-fold cross-validation with 95% confidence intervals (CIs).

Model	Training Data	Test Data					
		CelebAMask-HQ		FFHQ Glasses Det.		Mixed (All)	
		Mean	95% CI	Mean	95% CI	Mean	95% CI
YOLOv8xs	CelebAMask-HQ	0.98	0.97–1.00	0.95	0.94–0.97	0.95	0.95–0.96
	FFHQ Glasses Det.	0.99	0.99–0.99	0.98	0.98–0.98	0.98	0.98–0.98
	Mixed (All)	0.99	0.99–0.99	0.98	0.98–0.98	0.98	0.98–0.98
YOLOv8s	CelebAMask-HQ	0.98	0.98–0.98	0.95	0.94–0.96	0.96	0.95–0.96
	FFHQ Glasses Det.	0.99	0.97–1.00	0.98	0.98–0.98	0.98	0.98–0.98
	Mixed (All)	0.99	0.97–1.00	0.98	0.98–0.98	0.98	0.98–0.98
YOLOv8m	CelebAMask-HQ	0.98	0.98–0.98	0.94	0.94–0.95	0.95	0.95–0.96
	FFHQ Glasses Det.	0.99	0.97–1.00	0.98	0.98–0.98	0.98	0.98–0.98
	Mixed (All)	0.99	0.97–1.00	0.98	0.98–0.98	0.98	0.98–0.98
MobileNetV3s	CelebAMask-HQ	0.98	0.98–0.98	0.95	0.94–0.96	0.96	0.95–0.96
	FFHQ Glasses Det.	0.98	0.97–1.00	0.98	0.98–0.98	0.98	0.98–0.98
	Mixed (All)	0.99	0.99–0.99	0.98	0.98–0.98	0.98	0.98–0.98

The YOLOv8 backbones were pretrained on the COCO dataset, and MobileNetV3 was pretrained on the ImageNet dataset.

**Table A2:** Average Precision at the IoU = 0.75 (AP^IoU = 0.75^) baseline results for eyeglasses detection on CelebAMask-HQ; FFHQ Glasses Detection; and a mixed dataset, which combines both. Baseline models were trained on the training folds of different datasets (CelebAMask-HQ, FFHQ Glasses Detection, and mixed) and were tested on all the remaining folds of each dataset. The results are presented as the means of three-fold cross-validation with 95% confidence intervals (CIs).

Model	Training Data	Test Data					
		CelebAMask-HQ		FFHQ Glasses Det.		Mixed (All)	
		Mean	95% CI	Mean	95% CI	Mean	95% CI
YOLOv8xs	CelebAMask-HQ	0.88	0.79–0.98	0.88	0.85–0.90	0.88	0.85–0.90
	FFHQ Glasses Det.	0.93	0.85–1.00	0.95	0.92–0.97	0.94	0.92–0.96
	Mixed (All)	0.92	0.83–1.00	0.95	0.93–0.96	0.95	0.93–0.96
YOLOv8s	CelebAMask-HQ	0.89	0.81–0.98	0.89	0.88–0.90	0.89	0.88–0.90
	FFHQ Glasses Det.	0.92	0.83–1.00	0.95	0.93–0.96	0.95	0.93–0.96
	Mixed (All)	0.93	0.83–1.00	0.94	0.93–0.96	0.94	0.92–0.96
YOLOv8m	CelebAMask-HQ	0.89	0.81–0.98	0.88	0.87–0.89	0.88	0.87–0.89
	FFHQ Glasses Det.	0.93	0.83–1.00	0.95	0.94–0.96	0.94	0.93–0.95
	Mixed (All)	0.93	0.88–0.98	0.94	0.92–0.97	0.94	0.92–0.96
MobileNetV3s	CelebAMask-HQ	0.88	0.77–0.99	0.87	0.86–0.88	0.87	0.86–0.88
	FFHQ Glasses Det.	0.91	0.84–0.98	0.93	0.91–0.96	0.93	0.92–0.94
	Mixed (All)	0.92	0.80–1.00	0.93	0.91–0.94	0.92	0.92–0.93

The YOLOv8 backbones were pretrained on the COCO dataset, and MobileNetV3 was pretrained on the ImageNet dataset.

**Table A3:** Average Precision for the small objects (area < $300^{2}$) (AP^small^) baseline results for eyeglasses detection on CelebAMask-HQ; FFHQ Glasses Detection; and a mixed dataset, which combines both. The baseline models were trained on the training folds of different datasets (CelebAMask-HQ, FFHQ Glasses Detection, and mixed) and were tested on all the remaining folds of each dataset. The results are presented as the means of three-fold cross-validation with 95% confidence intervals (CIs).

Model	Training Data	Test Data					
		CelebAMask-HQ		FFHQ Glasses Det.		Mixed (All)	
		Mean	95% CI	Mean	95% CI	Mean	95% CI
YOLOv8xs	CelebAMask-HQ	0.75	0.70–0.81	0.76	0.74–0.78	0.76	0.74–0.78
	FFHQ Glasses Det.	0.79	0.73–0.85	0.82	0.81–0.83	0.82	0.81–0.82
	Mixed (All)	0.79	0.74–0.84	0.82	0.80–0.84	0.82	0.80–0.83
YOLOv8s	CelebAMask-HQ	0.77	0.72–0.82	0.77	0.77–0.78	0.77	0.77–0.78
	FFHQ Glasses Det.	0.80	0.75–0.86	0.84	0.83–0.84	0.83	0.82–0.84
	Mixed (All)	0.80	0.77–0.83	0.83	0.83–0.84	0.83	0.82–0.84
YOLOv8m	CelebAMask-HQ	0.79	0.73–0.85	0.78	0.76–0.79	0.77	0.77–0.78
	FFHQ Glasses Det.	0.80	0.74–0.87	0.84	0.82–0.86	0.84	0.82–0.86
	Mixed (All)	0.80	0.76–0.84	0.83	0.81–0.85	0.83	0.80–0.85
MobileNetV3s	CelebAMask-HQ	0.73	0.65–0.80	0.72	0.70–0.75	0.72	0.71–0.74
	FFHQ Glasses Det.	0.76	0.73–0.80	0.80	0.78–0.81	0.79	0.78–0.80
	Mixed (All)	0.78	0.76–0.80	0.78	0.77–0.80	0.78	0.77–0.80

The YOLOv8 backbones were pretrained on the COCO dataset, and MobileNetV3 was pretrained on the ImageNet dataset.

**Table A4:** Average Precision for the medium objects ($300^{2}$ < area < $340^{2}$) (AP^medium^) baseline results for eyeglasses detection on CelebAMask-HQ; FFHQ Glasses Detection; and a mixed dataset, which combines both. The baseline models were trained on the training folds of different datasets (CelebAMask-HQ, FFHQ Glasses Detection, and mixed) and were tested on all the remaining folds of each dataset. The results are presented as the means of three-fold cross-validation with 95% confidence intervals (CIs).

Model	Training Data	Test Data					
		CelebAMask-HQ		FFHQ Glasses Det.		Mixed (All)	
		Mean	95% CI	Mean	95% CI	Mean	95% CI
YOLOv8xs	CelebAMask-HQ	0.82	0.80–0.83	0.84	0.83–0.86	0.84	0.83–0.85
	FFHQ Glasses Det.	0.85	0.83–0.88	0.90	0.89–0.91	0.89	0.88–0.91
	Mixed (All)	0.85	0.80–0.89	0.90	0.89–0.91	0.89	0.89–0.89
YOLOv8s	CelebAMask-HQ	0.82	0.82–0.83	0.85	0.84–0.85	0.84	0.84–0.85
	FFHQ Glasses Det.	0.85	0.83–0.87	0.91	0.90–0.92	0.90	0.89–0.91
	Mixed (All)	0.86	0.83–0.89	0.91	0.89–0.92	0.90	0.89–0.92
YOLOv8m	CelebAMask-HQ	0.82	0.80–0.84	0.85	0.85–0.85	0.85	0.84–0.85
	FFHQ Glasses Det.	0.86	0.83–0.90	0.92	0.92–0.92	0.91	0.90–0.92
	Mixed (All)	0.86	0.85–0.88	0.91	0.90–0.91	0.90	0.89–0.90
MobileNetV3s	CelebAMask-HQ	0.80	0.77–0.83	0.81	0.79–0.82	0.81	0.80–0.82
	FFHQ Glasses Det.	0.83	0.82–0.85	0.88	0.87–0.88	0.87	0.87–0.87
	Mixed (All)	0.84	0.81–0.87	0.87	0.86–0.89	0.87	0.85–0.88

The YOLOv8 backbones were pretrained on the COCO dataset, and MobileNetV3 was pretrained on the ImageNet dataset.

**Table A5:** Average Precision for the large objects (area > $340^{2}$) (AP^large^) baseline results for eyeglasses detection on CelebAMask-HQ; FFHQ Glasses Detection; and a mixed dataset, which combines both. The baseline models were trained on the training folds of different datasets (CelebAMask-HQ, FFHQ Glasses Detection, and mixed) and were tested on all the remaining folds of each dataset. The results are presented as the means of three-fold cross-validation with 95% confidence intervals (CIs).

Model	Training Data	Test Data					
		CelebAMask-HQ		FFHQ Glasses Det.		Mixed (All)	
		Mean	95% CI	Mean	95% CI	Mean	95% CI
YOLOv8xs	CelebAMask-HQ	0.77	0.71–0.83	0.75	0.71–0.80	0.76	0.72–0.79
	FFHQ Glasses Det.	0.80	0.74–0.86	0.86	0.84–0.88	0.85	0.84–0.86
	Mixed (All)	0.80	0.74–0.87	0.86	0.85–0.88	0.86	0.85–0.86
YOLOv8s	CelebAMask-HQ	0.76	0.67–0.85	0.76	0.73–0.80	0.76	0.74–0.78
	FFHQ Glasses Det.	0.81	0.72–0.90	0.87	0.83–0.90	0.86	0.84–0.88
	Mixed (All)	0.81	0.73–0.89	0.86	0.85–0.88	0.86	0.84–0.87
YOLOv8m	CelebAMask-HQ	0.77	0.69–0.86	0.76	0.74–0.78	0.76	0.75–0.78
	FFHQ Glasses Det.	0.81	0.73–0.90	0.88	0.86–0.90	0.87	0.86–0.88
	Mixed (All)	0.81	0.76–0.86	0.87	0.83–0.90	0.86	0.83–0.89
MobileNetV3s	CelebAMask-HQ	0.74	0.67–0.82	0.74	0.70–0.77	0.74	0.71–0.76
	FFHQ Glasses Det.	0.78	0.72–0.84	0.83	0.80–0.86	0.83	0.80–0.85
	Mixed (All)	0.78	0.72–0.85	0.83	0.80–0.86	0.82	0.80–0.84

The YOLOv8 backbones were pretrained on the COCO dataset, and MobileNetV3 was pretrained on the ImageNet dataset.

**Table A6:** Average Recall for the small objects (area < $300^{2}$) (AR^small^) baseline results for eyeglasses detection on CelebAMask-HQ; FFHQ Glasses Detection; and a mixed dataset, which combines both. The baseline models were trained on the training folds of different datasets (CelebAMask-HQ, FFHQ Glasses Detection, and mixed) and were tested on all the remaining folds of each dataset. The results are presented as the means of three-fold cross-validation with 95% confidence intervals (CIs).

Model	Training Data	Test Data					
		CelebAMask-HQ		FFHQ Glasses Det.		Mixed (All)	
		Mean	95% CI	Mean	95% CI	Mean	95% CI
YOLOv8xs	CelebAMask-HQ	0.80	0.76–0.83	0.80	0.78–0.82	0.80	0.78–0.82
	FFHQ Glasses Det.	0.83	0.79–0.88	0.86	0.85–0.87	0.86	0.85–0.86
	Mixed (All)	0.82	0.78–0.87	0.85	0.84–0.87	0.85	0.84–0.86
YOLOv8s	CelebAMask-HQ	0.82	0.79–0.84	0.81	0.80–0.82	0.81	0.80–0.82
	FFHQ Glasses Det.	0.84	0.81–0.88	0.87	0.86–0.89	0.87	0.85–0.89
	Mixed (All)	0.84	0.82–0.86	0.87	0.86–0.88	0.87	0.86–0.88
YOLOv8m	CelebAMask-HQ	0.83	0.80–0.86	0.82	0.80–0.84	0.82	0.80–0.83
	FFHQ Glasses Det.	0.84	0.80–0.89	0.87	0.85–0.90	0.87	0.85–0.89
	Mixed (All)	0.84	0.82–0.86	0.86	0.83–0.90	0.86	0.83–0.90
MobileNetV3s	CelebAMask-HQ	0.77	0.72–0.82	0.76	0.73–0.79	0.76	0.74–0.79
	FFHQ Glasses Det.	0.80	0.80–0.81	0.83	0.81–0.85	0.83	0.81–0.85
	Mixed (All)	0.81	0.80–0.83	0.83	0.80–0.85	0.82	0.80–0.85

The YOLOv8 backbones were pretrained on the COCO dataset, and the MobileNetV3 was pretrained on the ImageNet dataset.

**Table A7:** Average Recall for the medium objects ($300^{2}$ < area < $340^{2}$) (AR^medium^) baseline results for eyeglasses detection on CelebAMask-HQ; FFHQ Glasses Detection; and a mixed dataset, which combines both. The baseline models were trained on the training folds of different datasets (CelebAMask-HQ, FFHQ Glasses Detection, and mixed) and were tested on all the remaining folds of each dataset. The results are presented as the means of three-fold cross-validation with 95% confidence intervals (CIs).

Model	Training Data	Test Data					
		CelebAMask-HQ		FFHQ Glasses Det.		Mixed (All)	
		Mean	95% CI	Mean	95% CI	Mean	95% CI
YOLOv8xs	CelebAMask-HQ	0.87	0.85–0.90	0.89	0.87–0.90	0.88	0.87–0.90
	FFHQ Glasses Det.	0.90	0.88–0.91	0.92	0.91–0.93	0.92	0.91–0.93
	Mixed (All)	0.89	0.86–0.93	0.92	0.92–0.93	0.92	0.92–0.93
YOLOv8s	CelebAMask-HQ	0.88	0.86–0.90	0.89	0.88–0.89	0.89	0.88–0.89
	FFHQ Glasses Det.	0.90	0.88–0.92	0.93	0.92–0.94	0.93	0.92–0.94
	Mixed (All)	0.91	0.88–0.93	0.93	0.92–0.95	0.93	0.92–0.94
YOLOv8m	CelebAMask-HQ	0.88	0.85–0.91	0.89	0.89–0.89	0.89	0.89–0.89
	FFHQ Glasses Det.	0.91	0.88–0.94	0.94	0.93–0.95	0.94	0.93–0.94
	Mixed (All)	0.90	0.88–0.92	0.93	0.92–0.94	0.93	0.92–0.94
MobileNetV3s	CelebAMask-HQ	0.85	0.82–0.89	0.85	0.85–0.86	0.85	0.85–0.86
	FFHQ Glasses Det.	0.88	0.85–0.90	0.90	0.90–0.91	0.90	0.90–0.90
	Mixed (All)	0.88	0.85–0.91	0.90	0.89–0.92	0.90	0.88–0.92

The YOLOv8 backbones were pretrained on the COCO dataset, and MobileNetV3 was pretrained on the ImageNet dataset.

**Table A8:** Average Recall for the large objects (area > $340^{2}$) (AR^large^) baseline results for eyeglasses detection on CelebAMask-HQ; FFHQ Glasses Detection; and a mixed dataset, which combines both. The baseline models were trained on the training folds of different datasets (CelebAMask-HQ, FFHQ Glasses Detection, and mixed) and were tested on all the remaining folds of each dataset. The results are presented as the means of three-fold cross-validation with 95% confidence intervals (CIs).

Model	Training Data	Test Data					
		CelebAMask-HQ		FFHQ Glasses Det.		Mixed (All)	
		Mean	95% CI	Mean	95% CI	Mean	95% CI
YOLOv8xs	CelebAMask-HQ	0.82	0.77–0.88	0.82	0.79–0.85	0.82	0.79–0.84
	FFHQ Glasses Det.	0.84	0.79–0.89	0.89	0.88–0.91	0.89	0.88–0.90
	Mixed (All)	0.84	0.79–0.90	0.90	0.88–0.91	0.89	0.88–0.90
YOLOv8s	CelebAMask-HQ	0.82	0.75–0.89	0.82	0.80–0.83	0.82	0.81–0.83
	FFHQ Glasses Det.	0.84	0.76–0.92	0.90	0.88–0.92	0.89	0.88–0.90
	Mixed (All)	0.85	0.80–0.90	0.90	0.88–0.91	0.89	0.88–0.90
YOLOv8m	CelebAMask-HQ	0.82	0.77–0.88	0.82	0.81–0.84	0.82	0.81–0.83
	FFHQ Glasses Det.	0.85	0.78–0.92	0.90	0.88–0.93	0.90	0.88–0.91
	Mixed (All)	0.85	0.80–0.90	0.90	0.88–0.92	0.89	0.88–0.90
MobileNetV3s	CelebAMask-HQ	0.80	0.73–0.87	0.79	0.77–0.81	0.79	0.78–0.80
	FFHQ Glasses Det.	0.82	0.75–0.88	0.87	0.85–0.89	0.86	0.85–0.87
	Mixed (All)	0.83	0.79–0.87	0.87	0.84–0.89	0.86	0.84–0.88

The YOLOv8 backbones were pretrained on the COCO dataset, and MobileNetV3 was pretrained on the ImageNet dataset.
